# Supplementary material for: Linear discriminant analysis reveals hidden patterns in NMR chemical shifts of intrinsically disordered proteins
Source: PLoS Comput Biol. 2022 Oct 6;18(10):e1010258. doi: 10.1371/journal.pcbi.1010258 (PMC9578625; doi:10.1371/journal.pcbi.1010258)
Supplement: S1 Text — We analyzed the performance of LDA, Quadratic Discriminant Analysis (QDA), K-Nearest Neighbours (KNN) and Support Vector Machines (SVM) to pick the best classification method for our research. We looked at specific performance parameters such as accuracy, sensitivity, specificity and consistency. (PDF) [file pcbi.1010258.s001.pdf]

Linear discriminant analysis reveals hidden patterns in NMR chemical shifts of intrinsically disordered proteins

Javier A. Romero[1◑], Paulina Putko[1◑], Mateusz Urbańczyk[2], Krzysztof Kazimierczuk[1*], Anna Zawadzka-Kazimierczuk[3*]

1 Centre of New Technologies, University of Warsaw, Warsaw, Poland
2 Institute of Physical Chemistry, Polish Academy of Sciences, Warsaw, Poland
3 Biological and Chemical Research Centre, Faculty of Chemistry, University of Warsaw, Warsaw, Poland

◑These authors contributed equally to this work.
*k.kazimierczuk@cent.uw.edu.pl, anzaw@chem.uw.edu.pl

LDA vs. other classification methods

In this section we compare the performance of LDA with that of Quadratic Discriminant Analysis (QDA), K-Nearest Neighbours (KNN) and Support Vector Machines (SVM). The same 17 proteins from the BMRB described in the main text were used for leave-one-out cross-validation of all mentioned methods (S1 Fig). KNN was implemented with votes from 9 nearest neighbours, *taxicab* metric to measure distance (L_1 norm) and using the inverse of the distance as weighting function for the votes. The SVM model was constructed using a one-versus-all coding design (for each binary learner, one class is positive and the rest are negative) and was implemented using polynomials of grade 3 as kernel function to map the data onto higher dimensions.

We further analyzed the classification performance of each method by computing their sensitivity and specificity for each amino acid residue type (S2 Fig), where:

$$Sensitivity = \frac{TruePositives}{TruePositives + FalseNegatives}$$
$$Specificity = \frac{TrueNegatives}{TrueNegatives + FalsePositives}$$

For a given amino acid type (e.g. alanine), True Positives are accurately classified residues of the given type (alanine classified as alanine) and False Positives denotes residues from other amino acid types classified into it (serine classified as alanine). Similarly, True Negatives denotes residues from other amino acid types not classified into the given type (serine classified as something else than alanine) and False Negatives are wrongly classified residues of the given type (alanine classified as serine). Sensitivity measures the true positive rate, or the ability of a method to correctly classify residues of a given amino acid type, while specificity measures the true negative rate, or the ability of a method to correctly disregard residues that are not of a given amino acid type [1]. The code for these tests was written in Matlab R2021a using the Statistics and Machine Learning toolbox.

A summary of the results obtained for LDA and all other classification methods is shown in S1 Table. LDA is superior in every aspect. And although performance values for other methods may come close to that of LDA, what makes LDA come through as the best choice for protein mapping is its reduced variance on classification accuracy.

In short, LDA is the best method to make consistent classification predictions across different IDPs.

References

1. Parikh R, Mathai A, Parikh S, Sekhar GC, Thomas R. Understanding and using sensitivity, specificity and predictive values. Indian journal of ophthalmology. 2008;56(1):45.
