# Supplementary material for: Linear discriminant analysis reveals hidden patterns in NMR chemical shifts of intrinsically disordered proteins
Source: PLoS Comput Biol. 2022 Oct 6;18(10):e1010258. doi: 10.1371/journal.pcbi.1010258 (PMC9578625; doi:10.1371/journal.pcbi.1010258)
Supplement: S2 Text — The results of LDA for the proteins from the training data obtained in the same way as Fig 7 (subset (iii)). (PDF) [file pcbi.1010258.s002.pdf]

Linear discriminant analysis reveals hidden patterns in NMR chemical shifts of intrinsically disordered proteins

Javier A. Romero[1◑], Paulina Putko[1◑], Mateusz Urbańczyk[2], Krzysztof Kazimierczuk[1*], Anna Zawadzka-Kazimierczuk[3*]

1 Centre of New Technologies, University of Warsaw, Warsaw, Poland
2 Institute of Physical Chemistry, Polish Academy of Sciences, Warsaw, Poland
3 Biological and Chemical Research Centre, Faculty of Chemistry, University of Warsaw, Warsaw, Poland

◑These authors contributed equally to this work.
*k.kazimierczuk@cent.uw.edu.pl, anzaw@chem.uw.edu.pl

LDA classification of proteins in the training set

All 17 proteins from the BMRB that compose the training set were assigned to further demonstrate the efficiency and accuracy of the LDA approach. In other words, the following Figures show the detailed assignment of each spin system that comprises the training set resulting from performing leave-one-out cross-validation as described in the main text. In all cases, chemical shift subset 3 from the main text was used (H^N, N, CO, Cα, Cβ, Hα, Hβ). Background color on spin system numbers represent the true amino acid type.
