# Supplementary material for: Linear discriminant analysis reveals hidden patterns in NMR chemical shifts of intrinsically disordered proteins
Source: PLoS Comput Biol. 2022 Oct 6;18(10):e1010258. doi: 10.1371/journal.pcbi.1010258 (PMC9578625; doi:10.1371/journal.pcbi.1010258)
Supplement: S3 Text — The performance of LDA approach was compared with the amino-acid recognition procedure of the TSAR program. [13] (PDF) [file pcbi.1010258.s003.pdf]

Linear discriminant analysis reveals hidden patterns in NMR chemical shifts of intrinsically disordered proteins

Javier A. Romero[1◕], Paulina Putko[1◕], Mateusz Urbańczyk[2], Krzysztof Kazimierczuk[1*], Anna Zawadzka-Kazimierczuk[3*]

1 Centre of New Technologies, University of Warsaw, Warsaw, Poland
2 Institute of Physical Chemistry, Polish Academy of Sciences, Warsaw, Poland
3 Biological and Chemical Research Centre, Faculty of Chemistry, University of Warsaw, Warsaw, Poland

◕These authors contributed equally to this work.
*k.kazimierczuk@cent.uw.edu.pl, anzaw@chem.uw.edu.pl

Comparison of LDA performance with TSAR amino-acid recognition procedure

The performance of LDA approach was compared with the amino-acid recognition procedure of the TSAR program[1,2]. Below, for each of the assigned spin systems, the recognized amino acid types were shown, for both of the approaches. In the case of LDA, the amino-acid symbols sizes correspond to the probabilities of these types. TSAR does not provide probabilities, it only excludes certain types, thus all the letters are of the same size.

References

1. Zawadzka-Kazimierczuk A, Koźmiński W, Billeter M. TSAR: A program for automatic resonance assignment using 2D cross-sections of high dimensionality, high-resolution spectra. Journal of Biomolecular NMR. 2012;54(1):81–95. doi:10.1007/s10858-012-9652-3.

2. Piai A, Gonnelli L, Felli IC, Pierattelli R, Kazimierczuk K, Grudziaz K, et al. Amino acid recognition for automatic resonance assignment of intrinsically disordered proteins. Journal of Biomolecular NMR. 2016;64. doi:10.1007/s10858-016-0024-2.
