# Supplementary material for: Linear discriminant analysis reveals hidden patterns in NMR chemical shifts of intrinsically disordered proteins
Source: PLoS Comput Biol. 2022 Oct 6;18(10):e1010258. doi: 10.1371/journal.pcbi.1010258 (PMC9578625; doi:10.1371/journal.pcbi.1010258)
Supplement: S2 Fig — Comparison of performance in classification for all 4 methods. (PDF) [file pcbi.1010258.s005.pdf]

# Linear discriminant analysis reveals hidden patterns in NMR chemical shifts of intrinsically disordered proteins

Javier A. Romero[1◑], Paulina Putko[1◑], Mateusz Urbańczyk[2], Krzysztof Kazimierczuk[1*], Anna Zawadzka-Kazimierczuk[3*]

**1** Centre of New Technologies, University of Warsaw, Warsaw, Poland
**2** Institute of Physical Chemistry, Polish Academy of Sciences, Warsaw, Poland
**3** Biological and Chemical Research Centre, Faculty of Chemistry, University of Warsaw, Warsaw, Poland

◑These authors contributed equally to this work.
*k.kazimierczuk@cent.uw.edu.pl, anzaw@chem.uw.edu.pl

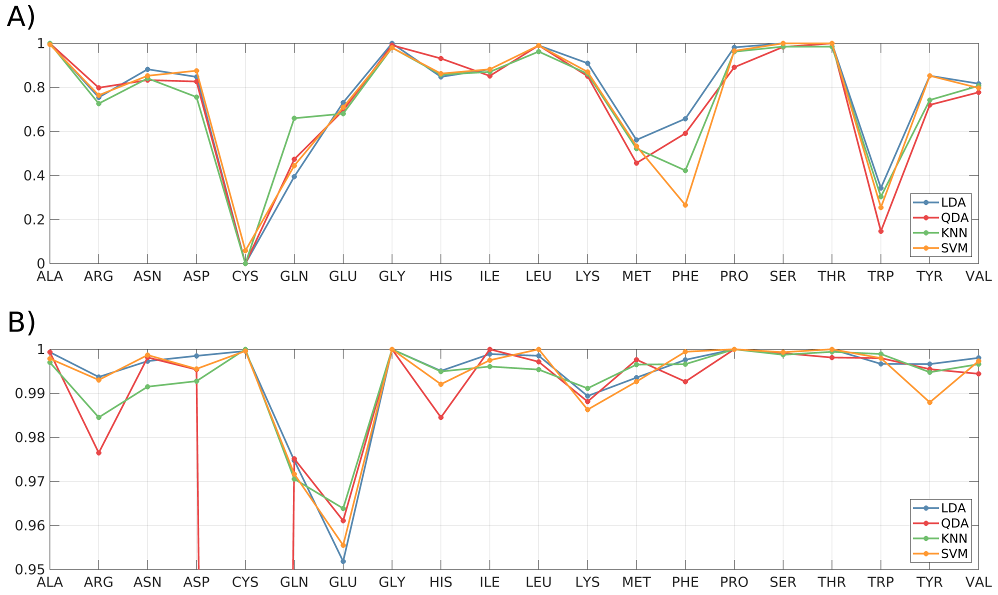


**S2 Fig** Comparison of classification performance for various methods. Results obtained from performing leave-one-out cross-validation using the 17 proteins from the training set and chemical shifts subset (iii). A) Sensitivity and B) specificity values for each amino acid type. All methods show a low performance for cysteine since there are only 5 residues of this type in the training set. In particular, this class needs to be removed for QDA since there are fewer samples than variables (which makes the class covariance matrix for this residue type singular).
