# Supplementary material for: Linear discriminant analysis reveals hidden patterns in NMR chemical shifts of intrinsically disordered proteins
Source: PLoS Comput Biol. 2022 Oct 6;18(10):e1010258. doi: 10.1371/journal.pcbi.1010258 (PMC9578625; doi:10.1371/journal.pcbi.1010258)
Supplement: S3 Fig — The results for all 17 proteins from the BMRB that compose the training set are shown, demonstrating the efficiency and accuracy of the LDA approach. (PDF) [file pcbi.1010258.s006.pdf]

Linear discriminant analysis reveals hidden patterns in NMR chemical shifts of intrinsically disordered proteins

Javier A. Romero[1◑], Paulina Putko[1◑], Mateusz Urbańczyk[2], Krzysztof Kazimierczuk[1*], Anna Zawadzka-Kazimierczuk[3*]

1 Centre of New Technologies, University of Warsaw, Warsaw, Poland
2 Institute of Physical Chemistry, Polish Academy of Sciences, Warsaw, Poland
3 Biological and Chemical Research Centre, Faculty of Chemistry, University of Warsaw, Warsaw, Poland

◑These authors contributed equally to this work.
*k.kazimierczuk@cent.uw.edu.pl, anzaw@chem.uw.edu.pl

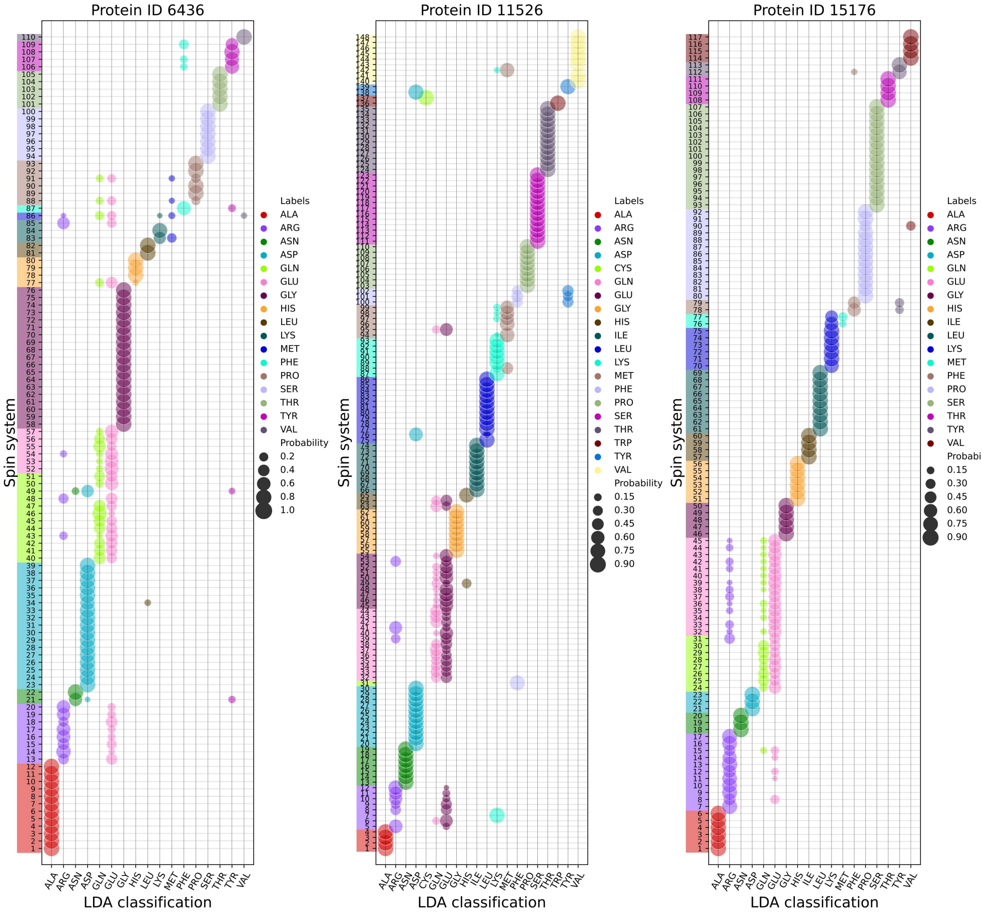

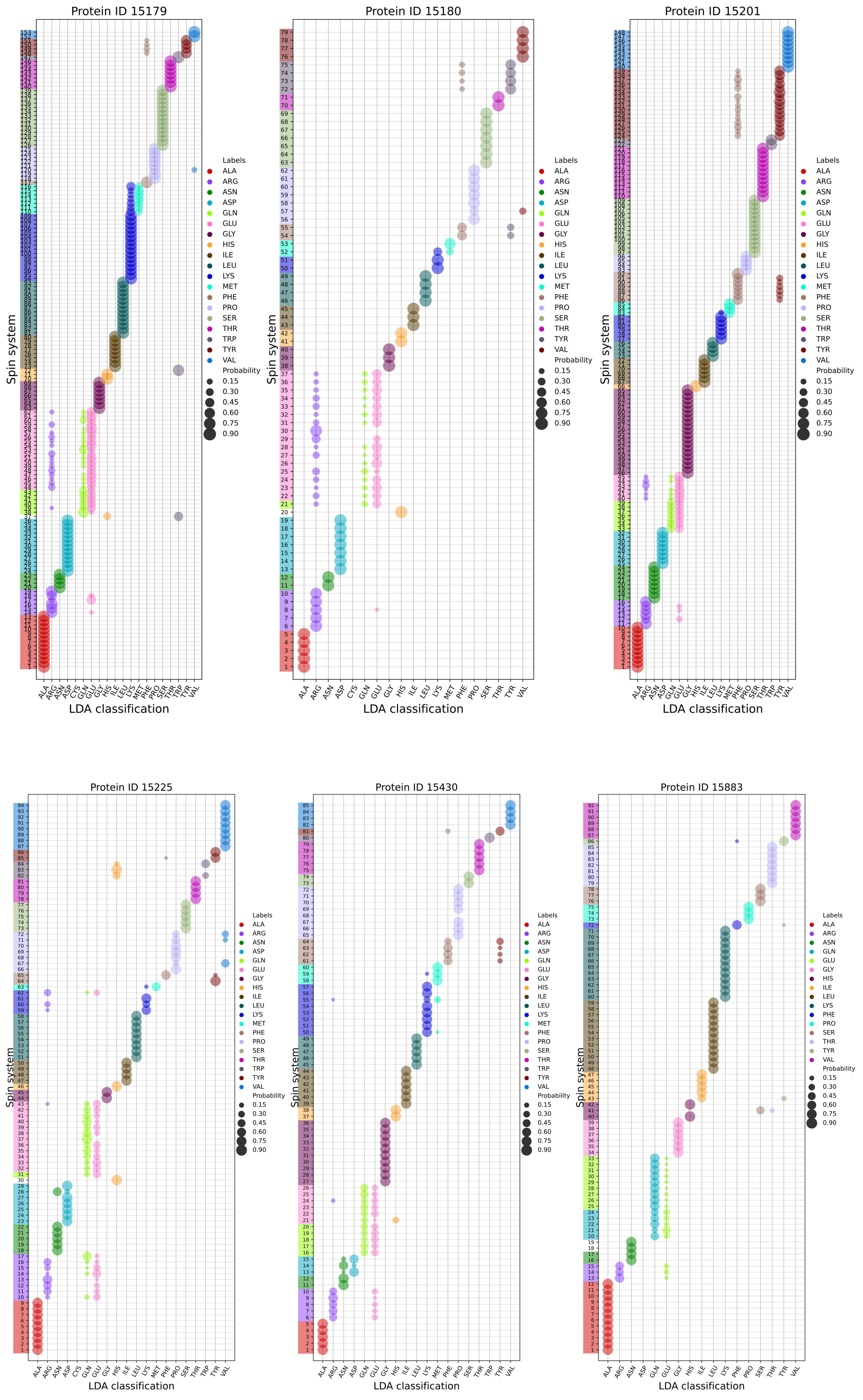
Protein ID 15179
Protein ID 15180
Protein ID 15201
Protein ID 15225
Protein ID 15430
Protein ID 15883
Spin system
LDA classification
Labels
ALA
ARG
ASN
ASP
GLN
GLU
GLY
HIS
ILE
LEU
LYS
MET
PHE
PRO
SER
THR
TRP
TYR
VAL
Probability
0.15
0.30
0.45
0.60
0.75
0.90

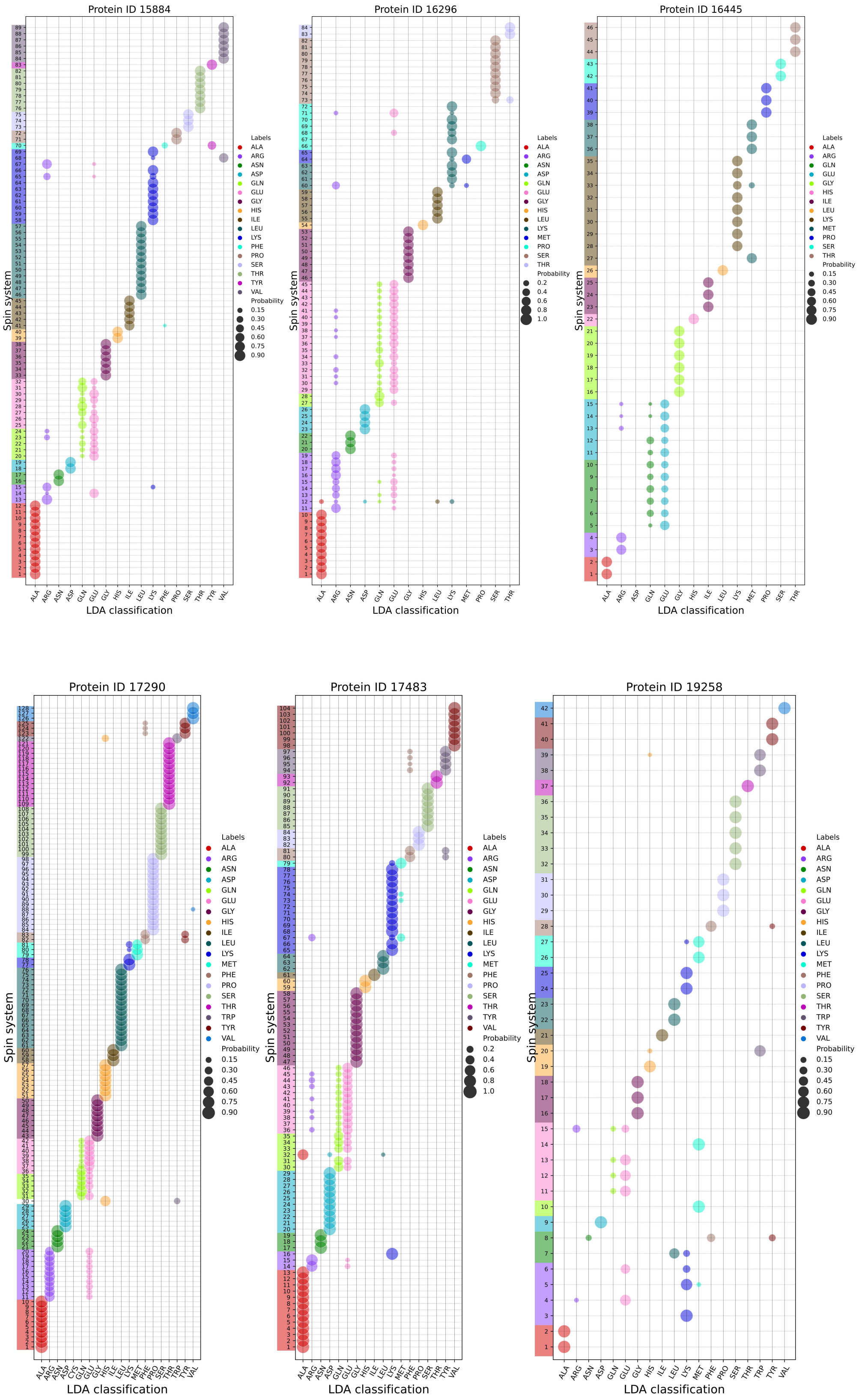
Protein ID 15884
Protein ID 16296
Protein ID 16445
Protein ID 17290
Protein ID 17483
Protein ID 19258
Spin system
LDA classification
Labels
Probability

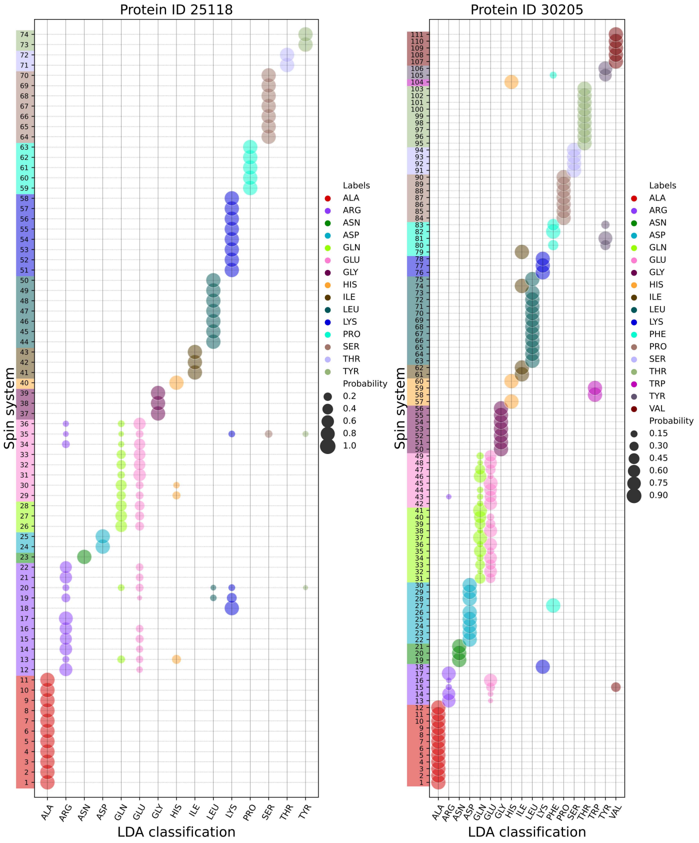

S3 Fig LDA results for proteins from the training set
