# Supplementary material for: Linear discriminant analysis reveals hidden patterns in NMR chemical shifts of intrinsically disordered proteins
Source: PLoS Comput Biol. 2022 Oct 6;18(10):e1010258. doi: 10.1371/journal.pcbi.1010258 (PMC9578625; doi:10.1371/journal.pcbi.1010258)
Supplement: S4 Fig — The results were shown for α-synuclein spin sytems. (PDF) [file pcbi.1010258.s007.pdf]

# Linear discriminant analysis reveals hidden patterns in NMR chemical shifts of intrinsically disordered proteins

Javier A. Romero[1◕], Paulina Putko[1◕], Mateusz Urbańczyk[2], Krzysztof Kazimierczuk[1*], Anna Zawadzka-Kazimierczuk[3*]

**1** Centre of New Technologies, University of Warsaw, Warsaw, Poland
**2** Institute of Physical Chemistry, Polish Academy of Sciences, Warsaw, Poland
**3** Biological and Chemical Research Centre, Faculty of Chemistry, University of Warsaw, Warsaw, Poland

◕These authors contributed equally to this work.
*k.kazimierczuk@cent.uw.edu.pl, anzaw@chem.uw.edu.pl

| TSAR | | Residue (atoms) | | LDA |
|---|---|---|---|---|
| N **D** F Y C | ← | **2D** (N, CO, $C_\alpha$, $C_\beta$, $H_\alpha$, $H_\beta$) | → | **D** |
| P **V** | ← | **3V** (N, CO, $C_\alpha$, $C_\beta$, $H_\alpha$, $H_\beta$) | → | **V** |
| D Y **F** C | ← | **4F** (N, CO, Cα, $C_\beta$, $H_\alpha$, $H_\beta$) | → | Y **F** |
| R Q E K **M** | ← | **5M** (N, CO, Cα, $C_\beta$, $H_\alpha$, $H_\beta$) | → | K **M** |
| P A S T G N D F Y I L R Q E V **K** M W H C | ← | **6K** (N, CO) | → | E **K** L |
| **G** | ← | **7G** (N, CO, Cα, $H_\alpha$) | → | **G** |
| **L** | ← | **8L** (N, CO, Cα, $C_\beta$, $H_\alpha$, $H_\beta$) | → | **L** |
| | | ⋮ | | |
| R Q **E** K M | ← | **13E** (N, CO, Cα, $C_\beta$, $H_\alpha$, $H_\beta$) | → | **E** Q |
| **G** | ← | **14G** (N, CO, Cα, $H_\alpha$) | → | **G** |
| P S T F Y I **V** W C | ← | **15V** (N, CO, Cα, $H_\alpha$) | → | **V** I |
| P **V** | ← | **16V** (N, CO, Cα, $C_\beta$, $H_\alpha$, $H_\beta$) | → | **V** |
| **A** | ← | **17A** (N, CO, $C_\alpha$, $C_\beta$, $H_\alpha$, Hβ) | → | **A** |
| **A** | ← | **18A** (N, CO, $C_\alpha$, $C_\beta$, $H_\alpha$, $H_\beta$) | → | **A** |
| **A** | ← | **19A** (N, CO, $C_\alpha$, $C_\beta$, $H_\alpha$, $H_\beta$) | → | **A** |
| R Q **E** K M | ← | **20E** (N, CO, $C_\alpha$, $C_\beta$, $H_\alpha$, $H_\beta$) | → | **E** Q |
| R Q E **K** M | ← | **21K** (N, CO, $C_\alpha$, $C_\beta$, $H_\alpha$, $H_\beta$) | → | **K** |
| **T** | ← | **22T** (N, CO, $C_\alpha$, $C_\beta$, $H_\alpha$, $H_\beta$) | → | **T** |
| R Q E **K** M | ← | **23K** (N, CO, $C_\alpha$, $C_\beta$, $H_\alpha$, $H_\beta$) | → | **K** |
| R **Q** E K M | ← | **24Q** (N, CO, $C_\alpha$, $C_\beta$, $H_\alpha$, $H_\beta$) | → | E **Q** |
| **G** | ← | **25G** (N, CO, $C_\alpha$, $H_\alpha$) | → | **G** |
| P **V** | ← | **26V** (N, CO, $C_\alpha$, $C_\beta$, $H_\alpha$, $H_\beta$) | → | **V** |
| **A** | ← | **27A** (N, CO, $C_\alpha$, $C_\beta$, $H_\alpha$, $H_\beta$) | → | **A** |
| R Q **E** K M | ← | **28E** (N, CO, $C_\alpha$, $C_\beta$, $H_\alpha$, $H_\beta$) | → | **E** Q |
| **A** | ← | **29A** (N, CO, $C_\alpha$, $C_\beta$, $H_\alpha$, $H_\beta$) | → | **A** |
| **A** | ← | **30A** (N, CO, $C_\alpha$, $C_\beta$, $H_\alpha$, $H_\beta$) | → | **A** |
| **G** | ← | **31G** (N, CO, $C_\alpha$, $H_\alpha$) | → | **G** |
| R E **K** M | ← | **32K** (N, CO, $C_\alpha$, $C_\beta$, $H_\alpha$, $H_\beta$) | → | **K** |
| **T** | ← | **33T** (N, CO, $C_\alpha$, $C_\beta$, $H_\alpha$, $H_\beta$) | → | **T** |

| TSAR | Residue (atoms) | LDA |
|---|---|---|
| **G** | **36G** (N, CO, $C_\alpha$, $C_\beta$, $H_\alpha$, $H_\beta$) | **G** |
| P **V** | **37V** (N, CO, $C_\alpha$, $C_\beta$, $H_\alpha$, $H_\beta$) | **V** |
| **L** | **38L** (N, CO, $C_\alpha$, $C_\beta$, $H_\alpha$, $H_\beta$) | **L** |
| D F **Y** C | **39Y** (N, CO, $C_\alpha$, $C_\beta$, $H_\alpha$, $H_\beta$) | **Y** F |
| P **V** | **40V** (N, CO, $C_\alpha$, $C_\beta$, $H_\alpha$, $H_\beta$) | **V** |
| **G** | **41G** (N, CO, $C_\alpha$, $H_\alpha$) | **G** |
| **S** | **42S** (N, CO, $C_\alpha$, $C_\beta$, $H_\alpha$, $H_\beta$) | **S** |
| R Q E **K** M | **43K** (N, CO, $C_\alpha$, $C_\beta$, $H_\alpha$, $H_\beta$) | **K** |
| **T** | **44T** (N, CO, $C_\alpha$, $C_\beta$, $H_\alpha$, $H_\beta$) | **T** |
| | ⋮ | |
| **G** | **47G** (N, CO, $C_\alpha$, $H_\alpha$) | **G** |
| P **V** | **48V** (N, CO, $C_\alpha$, $C_\beta$, $H_\alpha$, $H_\beta$) | **V** |
| P **V** | **49V** (N, CO, $C_\alpha$, $C_\beta$, $H_\alpha$) | **V** |
| W **H** C | **50H** (N, CO, $C_\alpha$, $C_\beta$, $H_\alpha$, $H_\beta$) | **H** |
| **G** | **51G** (N, CO, $C_\alpha$, $H_\alpha$) | **G** |
| P S T F Y I **V** W C | **52V** (N, CO, $C_\alpha$, $H_\alpha$) | **V** I |
| **A** | **53A** (N, CO, $C_\alpha$, $C_\beta$, $H_\alpha$, $H_\beta$) | **A** |
| **T** | **54T** (N, CO, $C_\alpha$, $C_\beta$, $H_\alpha$, $H_\beta$) | **T** |
| P **V** | **55V** (N, CO, $C_\alpha$, $C_\beta$, $H_\alpha$, $H_\beta$) | **V** |
| **A** | **56A** (N, CO, $C_\alpha$, $C_\beta$, $H_\alpha$, $H_\beta$) | **A** |
| R Q **E** K M | **57E** (N, CO, $C_\alpha$, $C_\beta$, $H_\alpha$, $H_\beta$) | **E** Q |
| R E **K** M | **58K** (N, CO, $C_\alpha$, $C_\beta$, $H_\alpha$, $H_\beta$) | **K** |
| **T** | **59T** (N, CO, $C_\alpha$, $C_\beta$, $H_\alpha$, $H_\beta$) | **T** |
| R Q E **K** M | **60K** (N, CO, $C_\alpha$, $C_\beta$, $H_\alpha$, $H_\beta$) | **K** |
| A S N D F Y I L R Q **E** K M W H C | **61E** (N, CO, $C_\alpha$, $H_\alpha$) | **E** K Q |
| R **Q** E K M | **62Q** (N, CO, $C_\alpha$, $C_\beta$, $H_\alpha$, $H_\beta$) | E **Q** |
| P **V** | **63V** (N, CO, $C_\alpha$, $C_\beta$, $H_\alpha$, $H_\beta$) | **V** |
| **T** | **64T** (N, CO, $C_\alpha$, $C_\beta$, $H_\alpha$, $H_\beta$) | **T** |

| TSAR | Residue | LDA |
|---|---|---|
| **N** D F C | **65N** (N, CO, $C_\alpha$, $C_\beta$, $H_\alpha$, $H_\beta$) | **N** |
| P **V** C | **66V** (N, CO, $C_\alpha$, $C_\beta$, $H_\alpha$, $H_\beta$) | **V** |
| **G** | **67G** (N, CO, $C_\alpha$, $H_\alpha$) | **G** |
| **G** | **68G** (N, CO, $C_\alpha$, $H_\alpha$) | **G** |
| **A** | **69A** (N, CO, $C_\alpha$, $C_\beta$, $H_\alpha$, $H_\beta$) | **A** |
| P **V** | **70V** (N, CO, $C_\alpha$, $C_\beta$, $H_\alpha$, $H_\beta$) | **V** |
| P **V** | **71V** (N, CO, $C_\alpha$, $C_\beta$, $H_\alpha$, $H_\beta$) | **V** |
| **T** | **72T** (N, CO, $C_\alpha$, $C_\beta$, $H_\alpha$, $H_\beta$) | **T** |
| **G** | **73G** (N, CO, $C_\alpha$, $C_\beta$, $H_\alpha$, $H_\beta$) | **G** |
| P **V** | **74V** (N, CO, $C_\alpha$, $C_\beta$, $H_\alpha$, $H_\beta$) | **V** |
| P S **T** F Y I V W C | **75T** (N, CO, $C_\alpha$, $H_\alpha$) | **T** v |
| **A** | **76A** (N, CO, $C_\alpha$, $C_\beta$, $H_\alpha$, $H_\beta$) | **A** |
| P **V** | **77V** (N, CO, $C_\alpha$, $C_\beta$, $H_\alpha$, $H_\beta$) | **V** |
| **A** | **78A** (N, CO, $C_\alpha$, $C_\beta$, $H_\alpha$, $H_\beta$) | **A** |
| R **Q** E K M | **79Q** (N, CO, $C_\alpha$, $C_\beta$, $H_\alpha$, $H_\beta$) | E **Q** |
| R E **K** M | **80K** (N, CO, $C_\alpha$, $C_\beta$, $H_\alpha$, $H_\beta$) | **K** |
| **T** | **81T** (N, CO, $C_\alpha$, $C_\beta$, $H_\alpha$, $H_\beta$) | **T** |
| P **V** | **82V** (N, CO, $C_\alpha$, $C_\beta$, $H_\alpha$, $H_\beta$) | **V** |
| R Q **E** K M | **83E** (N, CO, $C_\alpha$, $C_\beta$, $H_\alpha$, $H_\beta$) | **E** |
| **G** | **84G** (N, CO, $C_\alpha$, $C_\beta$, $H_\alpha$, $H_\beta$) | **G** |
| **A** | **85A** (N, CO, $C_\alpha$, $C_\beta$, $H_\alpha$, $H_\beta$) | **A** |
| **G** | **86G** (N, CO, $C_\alpha$, $H_\alpha$) | **G** |
| **S** | **87S** (N, CO, $C_\alpha$, $C_\beta$, $H_\alpha$, $H_\beta$) | **S** |
| **I** | **88I** (N, CO, $C_\alpha$, $C_\beta$, $H_\alpha$, $H_\beta$) | **I** |
| **A** | **89A** (N, CO, $C_\alpha$, $C_\beta$, $H_\alpha$, $H_\beta$) | **A** |
| **A** | **90A** (N, CO, $C_\alpha$, $C_\beta$, $H_\alpha$, $H_\beta$) | **A** |
| **A** | **91A** (N, CO, $C_\alpha$, $C_\beta$, $H_\alpha$, $H_\beta$) | **A** |
| **T** | **92T** (N, CO, $C_\alpha$, $C_\beta$, $H_\alpha$, $H_\beta$) | **T** |

| TSAR | | Residue | Atoms | | LDA |
|---|---|---|---|---|---|
| **G** | ← | **93G** | (N, CO, $C_\alpha$, $H_\alpha$,) | → | **G** |
| D **F** Y C | ← | **94F** | (N, CO, $C_\alpha$, $C_\beta$, $H_\alpha$, $H_\beta$) | → | **F** Y |
| P **V** | ← | **95V** | (N, CO, $C_\alpha$, $C_\beta$, $H_\alpha$, $H_\beta$) | → | **V** |
| R Q E **K** M | ← | **96K** | (N, CO, $C_\alpha$, $C_\beta$, $H_\alpha$, $H_\beta$) | → | **K** |
| R E **K** M | ← | **97K** | (N, CO, $C_\alpha$, $C_\beta$, $H_\alpha$, $H_\beta$) | → | **K** |
| N **D** F Y C | ← | **98D** | (N, CO, $C_\alpha$, $C_\beta$, $H_\alpha$, $H_\beta$) | → | **D** |
| A S N D F Y L R **Q** E K M W H C | ← | **99Q** | (N, CO, $C_\alpha$, $H_\alpha$,) | → | **Q** E K L |
| **L** | ← | **100L** | (N, CO, $C_\alpha$, $C_\beta$, $H_\alpha$, $H_\beta$) | → | **L** |
| **G** | ← | **101G** | (N, CO, $C_\alpha$, $C_\beta$, $H_\alpha$, $H_\beta$) | → | **G** |
| R E **K** M | ← | **102K** | (N, CO, $C_\alpha$, $C_\beta$, $H_\alpha$, $H_\beta$) | → | **K** |
| **N** D F Y C | ← | **103N** | (N, CO, $C_\alpha$, $C_\beta$, $H_\alpha$, $H_\beta$) | → | **N** |
| R Q **E** K M | ← | **104E** | (N, CO, $C_\alpha$, $C_\beta$, $H_\alpha$, $H_\beta$) | → | **E** Q |
| R Q **E** K M | ← | **105E** | (N, CO, $C_\alpha$, $C_\beta$, $H_\alpha$, $H_\beta$) | → | **E** Q |
| **G** | ← | **106G** | (N, CO, $C_\alpha$, $H_\alpha$) | → | **G** |
| | | ⋮ | | | |
| **G** | ← | **111G** | (N, CO, $C_\alpha$, $H_\alpha$) | → | **G** |
| **I** | ← | **112I** | (N, CO, $C_\alpha$, $C_\beta$, $H_\alpha$, $H_\beta$) | → | **I** |
| **L** | ← | **113L** | (N, CO, $C_\alpha$, $C_\beta$, $H_\alpha$, $H_\beta$) | → | **L** |
| R Q **E** K M | ← | **114E** | (N, CO, $C_\alpha$, $C_\beta$, $H_\alpha$, $H_\beta$) | → | **E** |
| N **D** F Y C | ← | **115D** | (N, CO, $C_\alpha$, $C_\beta$, $H_\alpha$, $H_\beta$) | → | **D** |
| | | ⋮ | | | |
| N **D** F Y C | ← | **121D** | (N, CO, $C_\alpha$, $C_\beta$, $H_\alpha$, $H_\beta$) | → | **D** |
| **N** D F Y C | ← | **122N** | (N, CO, $C_\alpha$, $C_\beta$, $H_\alpha$, $H_\beta$) | → | **N** |
| R Q **E** K M | ← | **123E** | (N, CO, $C_\alpha$, $C_\beta$, $H_\alpha$, $H_\beta$) | → | **E** Q |
| **A** | ← | **124A** | (N, CO, $C_\alpha$, $C_\beta$, $H_\alpha$, $H_\beta$) | → | **A** |
| S T D F **Y** I L R Q E V K M W H C | ← | **125Y** | (N, CO, $C_\alpha$, $H_\alpha$) | → | **Y** S F |
| R Q **E** K M | ← | **126E** | (N, CO, $C_\alpha$, $C_\beta$, $H_\alpha$, $H_\beta$) | → | **E** |

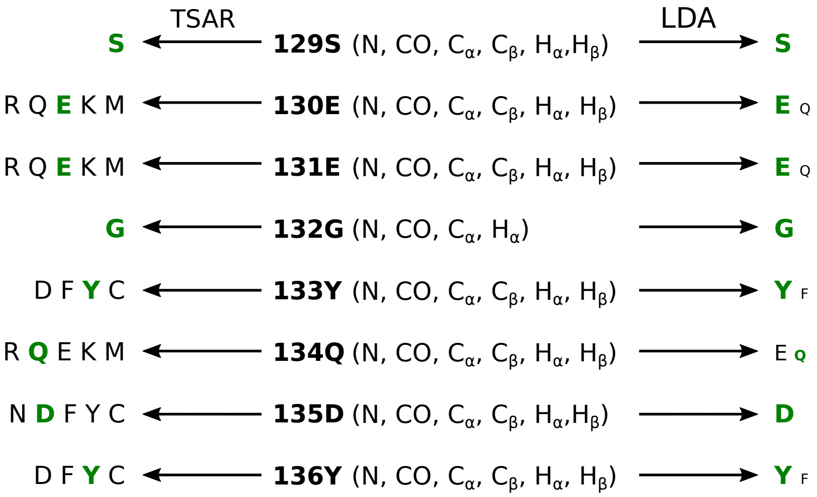


**S4 Fig** Comparison of LDA performance with TSAR amino-acid recognition procedure, for spin systems of the $\alpha$-synuclein protein.
