# Supplementary material for: Linear discriminant analysis reveals hidden patterns in NMR chemical shifts of intrinsically disordered proteins
Source: PLoS Comput Biol. 2022 Oct 6;18(10):e1010258. doi: 10.1371/journal.pcbi.1010258 (PMC9578625; doi:10.1371/journal.pcbi.1010258)
Supplement: S1 Table — Values of analyzed classification performance parameters are given for each method. (PDF) [file pcbi.1010258.s008.pdf]

Linear discriminant analysis reveals hidden patterns in NMR chemical shifts of intrinsically disordered proteins

Javier A. Romero[1◑], Paulina Putko[1◑], Mateusz Urbańczyk[2], Krzysztof Kazimierczuk[1*], Anna Zawadzka-Kazimierczuk[3*]

1 Centre of New Technologies, University of Warsaw, Warsaw, Poland
2 Institute of Physical Chemistry, Polish Academy of Sciences, Warsaw, Poland
3 Biological and Chemical Research Centre, Faculty of Chemistry, University of Warsaw, Warsaw, Poland

◑These authors contributed equally to this work.
*k.kazimierczuk@cent.uw.edu.pl, anzaw@chem.uw.edu.pl

Parameter	LDA	QDA	KNN	SVM
mean accuracy [%]	89.51	87.41	87.86	88.16
variance	35.80	97.43	73.99	64.29
mean sensitivity	0.773	0.741	0.747	0.748
mean specificity	0.994	0.943	0.993	0.993

S1 Table Summary of classification performances. Mean accuracies are weighted by the number of residues in the test protein. Variances were computed from the values displayed in Fig. S1. Mean sensitivities and specificities were computed from the values shown in Fig. S2
