# Supplementary material for: Linear discriminant analysis reveals hidden patterns in NMR chemical shifts of intrinsically disordered proteins
Source: PLoS Comput Biol. 2022 Oct 6;18(10):e1010258. doi: 10.1371/journal.pcbi.1010258 (PMC9578625; doi:10.1371/journal.pcbi.1010258)
Supplement: S2 Table — Sample conditions of the training data sets, as reported in BMRB entries. (PDF) [file pcbi.1010258.s009.pdf]

Linear discriminant analysis reveals hidden patterns in NMR chemical shifts of intrinsically disordered proteins

Javier A. Romero[1◕], Paulina Putko[1◕], Mateusz Urbańczyk[2], Krzysztof Kazimierczuk[1*], Anna Zawadzka-Kazimierczuk[3*]

1 Centre of New Technologies, University of Warsaw, Warsaw, Poland
2 Institute of Physical Chemistry, Polish Academy of Sciences, Warsaw, Poland
3 Biological and Chemical Research Centre, Faculty of Chemistry, University of Warsaw, Warsaw, Poland

◕These authors contributed equally to this work.
*k.kazimierczuk@cent.uw.edu.pl, anzaw@chem.uw.edu.pl

Protein	Size [kDa]	concentration[mM]	T[K]	pH	ionic strength[mM]
6436	12.05*	not available	298	6	not available
11526	17	0.2	303	6.5	not available
15176	13.54*	2	298	5.5	100
15179	17.96	0.75	298	6.5	70
15180	9.23	0.67	298	6.5	70
15201	16.39*	3	288	6.5	not available
15225	11.86*	0.6	298	6.5	50
15430	13.29	0.05/0.09	298	4	0
15883	9.98*	1	298.2	2	not available
15884	9.98*	1	298.2	3.8	120
16296	9.16*	1	298	7	120
16445	5.38*	1	300	6	10
17290	15.38*	1	298	6	not available
17483	11.69*	0.2	298	7	100
19258	5.04	1.7	291	7.5	50
25118	9.19*	0.5	313	6.2	120
30205	12.35	1.4	298	6.6	200

S2 Table. Sample conditions of the training data sets, as reported in BMRB entries. Protein sizes marked with (*) were calculated using `https://www.bioinformatics.org/sms/prot_mw.html`
