# Supplementary material for: Linear discriminant analysis reveals hidden patterns in NMR chemical shifts of intrinsically disordered proteins
Source: PLoS Comput Biol. 2022 Oct 6;18(10):e1010258. doi: 10.1371/journal.pcbi.1010258 (PMC9578625; doi:10.1371/journal.pcbi.1010258)
Supplement: Attachment — Submitted filename: answer to reviewers.pdf [file pcbi.1010258.s010.pdf]

Dear Editors,

We are very grateful to the Reviewers for sharing their thoughts and opinions on the article. Below you will find our answers to each and everyone of their comments and suggestions. We start with some general remarks that are crucial for further discussions.

Sincerely,
Krzysztof Kazimierczuk and Anna Zawadzka-Kazimierczuk

General remarks:

1. We apologize for not providing a link to the GitHub repository containing the code. We made the code available even before the initial submission, but we forgot to add the relevant link. The corrected version of the paper contains this statement in the text:

   "The python code used for LDA analysis along with NMR data of the α-synuclein IDP and all input files needed to reproduce the results shown in Fig. 7 are readily available at a public GitHub repository [30]."

   and in the abstract:

   "The code is freely available https://github.com/gugumatz/LDA-for-mapping-IDPs. The data is available at zenodo.org (10.5281/zenodo.7032142)."

   Importantly, the current version of the code has a new feature - it allows to filter out "impossible chains" that do not match with any sequence of aa-residues in a protein under consideration.

2. We would like to emphasize that the described method <u>is not</u> another sequential assignment technique. Because of that, it is difficult to compare it with sequential assignment programs. LDA identifies amino acid residues based on chemical shifts, so it improves just one step of the sequential assignment procedure (the chain mapping). On the other hand, LDA can be applied to peak list transfer described as "the Application 2", and this application does not necessarily require data on sequential connectivities. Our proposed optimal subset of chemical shifts (subset ii) does not contain them and alone would be useless for sequential assignment.

3. We realized that we accidentally put one folded protein into the training data set (entry 6869). We removed this entry in the revised version of the paper and modified all figures accordingly.

Reviewer 1:

Remark: Provided that the software is made available (which seems not to be the case now), I propose publication after (very) minor revision.

Answer: We added a link (see also a general remark #1 above).

Minor points:

Remark: Author summary, p.1: NMR is one of the methods for IDP research, rather than “the method”.

Answer: We are grateful for this comment. We corrected the text.

Remark: Methods (or Results): Describe the preparation of spin systems, in particular, how ambiguities have been handled/resolved.

Answer: We added a following fragment into Materials and Methods section:

“Spin systems were formed by gathering data from the peaks appearing on cross-sections corresponding to individual basis spectrum peaks. In the case of overlap of the basis spectrum peaks, when on a given cross-section appeared peaks from more than one spin system, the discrimination of spin systems was performed according to recommendations described in [13]: peaks were regarded as belonging to a cross-section on which they had higher absolute intensity. Using 3D HNCO (instead of ^{15}N-HSQC) as a basis spectrum made this approach more efficient; amide proton and nitrogen chemical shifts were determined more accurately. Thus, the overlapping cross-sections were more shifted than they would be if 2D basis spectrum was used.“

Remark: p. 5: The results of the comparison of four classification methods in the SI is hardly discussed in the main paper

Answer: We are grateful for pointing this out. Yet, we would not like to extend the discussion too much in the main manuscript. Thus, we just added a following text:

“Of note, other classification methods were also tested (as shown in SI), but LDA obtained the highest performance scores among all of them.”

Remark: p. 6: ‘(see Section)’ misses the section number.

p. 7: ‘described in Section ‘ misses the section number.

Answer: Lack of section numbering made referencing difficult. But we corrected it by using section titles.

Remark: p. 8: phenyloalanine —> phenylalanine (2x)

Answer: Corrected

Remark: p. 9, Application 1: Again, describe how spin systems are formed

Answer: Text added, see above.

Remark: p. 9, caption of Fig. 8: From which protein(s) are the examples shown?

Answer: We are grateful for pointing this out. Explanation added (alpha-synuclein).

Remark: P. 10: ’16 resonances in the region of interest’: Is the situation similar the other residues?

Answer: We provided (in Zenodo) the whole spectrum with BMRB and experimental peaks labeled. The data is mentioned in a caption to Figure 9.

Remark: Ref. 35 Wu K —> Wüthrich K

Answer: Corrected

Remark: Ref. 36 Orekhov VYVY —> Orekhov VY

Answer: Corrected

Remark: Fig. 6: What are the outliers at w(HB) = 0.6-0.8 ppm?

Answer: The mentioned outliers are: ASN30, ARG18 and PRO 41 from BMRB protein ID 19258. The authors of the original work do not discuss the exceptionally low Hβ shifts.

Reviewer 2:

Remark: 1. The authors somehow failed to demonstrate that the approach have any advantages over the use of the 'traditional' chemical shift connectivity methods along that are very successful to assign small and well-behaving IDPs as 1 mM aSyn. The importance of the work would be significantly enhanced if the author could demonstrate that the proposed algorithm improves accuracy and/or time-efficiency for more complex IDPs; for examples, IDPs with limited/sparse chemical shift connectivities (e.g., due to low sample concentrations or poor data quality) or/and with significant peak overlapping (e.g. 300+ a.a. IDPs).

Answer: We are grateful for this comment, but we have to emphasize that the method is not an operative resonance assignment program and thus it cannot be compared to “traditional chemical shift connectivity methods”. Currently, it is an algorithm that supports one step of the assignment procedure, having the potential to be included into automatic assignment programs in the future. We added the fragment clarifying this fact at the end of the Introduction section:

“It is worth mentioning that this method can be integrated into automatic assignment programs to facilitate the mapping step. Yet, it is not the only application for LDA, as shown in the results section.”

Regarding the application of LDA to other systems, we tested the approach on all proteins from the training data (using the ‘leave-one-out’ method). Now we added the charts presenting all these results in the Supplementary Information, showing that the high efficiency is not limited to aSyn. The set includes proteins of sizes up to 17.96 kDa and concentrations down to 50 μM. Of course, for these proteins, we did not perform the sequential connectivity step, as the proposed algorithm does not provide a solution to this problem. Still, we did face some difficulties resulting from experimental limitations, like missing peaks leading to gaps in chemical shift information.

Also, we would like to point out that aSyn provides a very crowded spectrum for a protein of this size. We intentionally used 4D experiments (instead of 5D providing better-resolved spectra) to demonstrate how to deal with the assignment of short chains of spin systems. As we write in the Practical Recommendations section, for very large IDPs we would consider a different approach, i.e. using the assigned part of the data for training and the remaining part for testing. We believe that we will be able to show this in future studies.

Remark: 2. The figure 5 clearly demonstrated that the accuracy of amino type predictions is <70% for 7 a.a. types out of 20, even if all seven chemical shifts were used for the analysis. The authors should provide specific examples to demonstrate how this (sometimes inaccurate) information could be incorporated into the assignment pipeline and how the use of this information would benefit the accuracy and/or efficiency the assignment process.

Answer: The way how "inaccurate information" can still be useful has been demonstrated in the section "Application 1". The current (improved) code automatically discards impossible chains, based on the output of the LDA analysis and an additional input file containing the chains of spin systems. The following text has been added to section Application 1: Mapping spin-system chains:

"Optionally, the LDA analysis can be followed by filtering the results using an amino-acid sequence of the protein under consideration. Filtering "impossible" chains can be done automatically by using the output of the LDA analysis. First, for each chain a number of amino acid sequences are formed, which rise from all the combinations of amino acid types that LDA predicts as probable for each spin system in the chain. Then, combinations which are not present in the sequence of the test protein are discarded. This procedure is included in the code provided in the GitHub repository as an optional feature: if an input file containing the spin systems chains is given as input, then the code will give an extra spreadsheet as output detailing all possible amino acid sequences for each chain, their probabilities and the discarded combinations. In the examples below we did use this option."

Examples of using "inaccurate information" are shown in Figure 8 and its description in the section "Application 1", e.g. for panel C) we write:

"Sometimes, as in Fig. 8C), the probability corresponding to the correct amino acid type is not the highest one. Fortunately, we can make the correct choice based on a protein sequence that lets us rule out "impossible" chains, even if the LDA implies that they are the most probable ones. Notably, unambiguous manual mapping of the chain in Fig. 8C) is not possible, as only one characteristic residue (glycine) is present."

Also, the following text was added to the last point of the Practical recommendations section:

"Automatic filtering of impossible chains is incorporated as an option in our code."

However, we would like to point out that even if accuracy is less than 70%, LDA typically shows the largest probability for the correct AA type, except for GLN and CYS. Yet, in the case of CYS this behavior can be explained due to the low number of samples in the training set (only 6 such residues among all the selected proteins) hindering appropriate training of the algorithm.

Remark: 3. The proposed methods obviously relies on the availability (particularly CB) chemical shifts for individual spin systems. However, for the larger (100+ a.a.) IDPs the unambiguous assignments of 3D and 4D peaks to a specific spin systems often became a major issue due to peak overlapping in NH 2D. The authors should discuss how this would affect the accuracy of the prediction.

Answer: We are grateful for this comment. Indeed, the issue deserves extended discussion. We added the following text into the Practical Recommendations section, below the suggestion to use NUS:

"The proper separation of NH resonances is particularly important since it affects the proper determination of the most differentiating chemical shifts (Cβ, Hβ) and formation of spin systems. Besides resolution-enhancement by NUS, one may solve the problem by increasing the experiment dimensionality - acquisition of 5D HabCabCONH would allow separating the spin systems using triples of frequencies (H^N_i, N_i and CO_{i-1}) which significantly reduces the overlap problem.[25,26] Another approach can be the application of methods based on ^{13}C detection.[42] On the other hand, the ambiguous cases that result from peak overlap can be easily detected and not taken into consideration. Thus, the situation is not as "dangerous" as e.g. for the sequential assignment."

Remark: 4. In Application 2, the authors should show the accuracy of assignment transfer for all peaks in the spectra rather than for a small regions as well as compare it with the accuracy of assignment transfer obtained using only connectivity information from the 4D HabCac(CO)NH spectrum. Moreover, I expect that for aSyn, the assignments for the majority of resonances can be unambiguously transfered using connectivities obtained from a simpler (and more sensitive) 3D HNCA experiment, while CB chemical shift would be critical for the LDA approach. The authors should provide example(s) that clearly demonstrates that the information about a.a. types (in addition to connectivity information obtained from the same experiment) would result in more accurate/efficient assignment transfer.

Answer: We are grateful for this comment. We added a file with the whole spectrum labeled with BMRB and experimental peaks to Zenodo (new DOI: 10.5281/zenodo.7032142). Yet, we would like to clarify a clear misunderstanding - the HabCab(CO)NH spectrum does not show connectivities, and thus we cannot "compare with the accuracy of assignment transfer obtained using only connectivity information from the 4D HabCab(CO)NH spectrum". Although we agree that 3D HNCA could be used for transfer based on establishing connectivities, it is not comparable to the LDA method. The latter provides the result with far less effort (only peak-picking, no sequential assignment needed). Although it is just an estimation of probabilities, we show that for the peak list transfer the information is typically sufficient due to usually quite limited options of possible peak shifts.

Remark: 5. The authors should provide detailed information about the training protein set they used, including protein sizes, a.a. composition, experimental conditions (particularly, pH, temperature, referencing method).

Answer: We added a new Table into SI containing experimental conditions. The amino acid composition is shown in the revised Figure 3 of the main text.

Reviewer #3:

Remark: (1) Given that this new LDA approach seems to have most utility in the resonance assignment procedure, the authors should compare the performance of their LDA approach with some other automatic resonance assignment software (e.g., MARS, TSAR, etc.). An ideal test scenario would be a performance comparison on alpha-synuclein given the same input chemical shifts (e.g., from the single 4D HabCab(CO)NH). Also, it would also be interesting to compare their performance of e.g. MARS or TSAR or AUTO-ASSIGN on the BMRB benchmark of 18 proteins with the results from the LDA approach.

Answer: As stated in general remark #2, LDA is not a sequential/automatic assignment procedure, so we cannot directly compare LDA with MARS, TSAR etc. In fact, it supports one of the assignment steps (i.e. chain mapping), and this step should be compared between the programs. However, most of the programs do not show such intermediate results; they rather show final assignment results. The only program that provides such data is TSAR, and we added (to the Supplementary Information) the comparison of the amino-acid type recognition (of each spin system separately) by TSAR and LDA.

Remark: --The main strength of the LDA approach, as I see it, is the LDA classification percentages (e.g., Figure 5 and Figure 7), which provides the user with more than one option in cases of low confidence. Thus, other software that only return one solution may yield inaccurate solutions whereas the LDA approach would very easily identify some ambiguity in the assignment solution space, which could most likely be easily resolved (as shown in Figure 8). Thus, it is important to show that the LDA approach provides new information relative to other state-of-the-art automatic assignment approaches. -

Answer: As stated in the previous answer, the only program which provides direct information on the mapping step is TSAR, and we made the (limited) comparison with it. However, the complete comparison (chain mapping of all protein chains, taking into account the already occupied sites etc.) would only be possible after incorporating LDA into some assignment software that we hope for in future.

Remark: (2) In Figure 8 (p. 9 of the text), the authors discuss how the LDA approach provides probability distributions in which one class (AA type) may not dominate (i.e., there is assignment ambiguity). The authors then state that the protein sequence can be used to rule out impossible solutions that cannot be consistent with the proposed assignments and the primary structure.

--I wonder if the authors could incorporate this manual step into the automatic analysis procedure? For example, if this manual calculation could be included as an automatic post-analysis step that follows the initial LDA classification, it would be helpful to the user. Because I fear that uninformed users will simply take the highest assignment probability as the “answer” and not check for compatibility with the primary structure/chemical shifts.

Answer: This is a very good point. Automatic filtering of impossible chains has now been added to the code. This new feature is mentioned in several sections of the revised article.

Remark: --If this analysis of probability distributions could be coded as consistency check with the primary structure/chemical shifts, it would be a nice extension of the work to ensure that the assignment ambiguity does not lead to errors but rather is exploited by the program to circumvent errors. Of course, there will be situations where the probability distribution analysis followed by a post-processing step of consistency with primary structure (which the authors currently perform manually on a case-by-case basis) cannot return an unambiguous correct result. In these cases, the LDA approach shines because it has informed the user that multiple solutions fit the data equally well and there may be the need to collect additional NMR data or to manually inspect the data very closely. Having the two steps combined into one automatic approach (i.e., LDA analysis + inspection of consistency with primary structure) would further strengthen the confidence in the results, knowing that the consistency check may be able to prevent erroneous interpretation of the results by the users.

Answer: Please check the previous answer.

(3) The authors assume one normal distribution for the chemical shift of a given spin in a given residue. And the authors further assume that the input chemical shifts in the benchmark would be representative of the underlying chemical shift distribution in the protein under investigation. However, the authors' assumption of a single normal distribution may be problematic in the scenario in which the IDP under investigation has significant residual structure, meaning the distribution would have to cover +/-4 ppm. Residual secondary structure occurs frequently in IDPs, and a quick browsing of the BMRB benchmark used by the authors shows that BMRB ID 11526 has nearly significant alpha-helical secondary structure between residues ~60-78, reaching a maximum value of ~50% near residue 70 (https://link.springer.com/). Another transient helix is found with secondary 13CA shifts of ~2 ppm in Darrp-32 (https://pubs.acs.org/doi/10.) from BMRB ID 15176 in the benchmark. Finally, in BMRB 15179 of the benchmark, there are at least 3 transient helices, the most C-terminal of which has secondary 13CA values near 4 ppm (from the same paper) suggesting near complete formation of the helix.

Answer: We are grateful for pointing this out. As stated in Conclusions: "In our experience, it is easy to obtain a training set from the BMRB with only coarse filtering—that is to say, using proteins marked as "unfolded", "unstructured" or "disordered".

We have been surprised by how tolerant the method is to little outliers in the training data. Of course, these regions could be manually removed and perhaps better-tailored training data could be prepared. We did not remove any residues for the purpose of showing the robustness of the method. Yet, the details mentioned by the reviewer are very interesting, and we added them into the discussion of the robustness of the method (Practical Recommendations):

"In fact, the training data contained small regions with residual structure: α-helical (up to 50%) for residues 60-78 in BMRB data set 11526, similarly long (19 residues) α-helical linker in set 15176 and several transient helices in data set 15179 (one of them with secondary shifts of Cα of up to 4 ppm, suggesting complete formation of the helix)."

Remark: 3a. Given these large secondary CA values (and presumably correspondingly large HA, HB, CB, CO secondary shifts going in the appropriate directions), could the authors comment on how they expect deviations from random coil character to affect the performance of the LDA classification?

Answer: We are grateful for this suggestion. We checked how disorder level, amino acid composition and other factors are related to the efficiency of LDA classification for the proteins from the training set. The relations are not simple, which justifies the title ("hidden patterns"). Please check the answer to Minor Remark 5 below.

Remark: 3b. I wonder if there is a correlation between the errors produced by LDA analysis and the secondary CA or CB shifts? That is to say, have the authors noticed that the LDA classifier fails in situations where there is significant residual structure? This might be expected if the majority of residues in the benchmark are random coil whereas a small minority adopt transient secondary structure.

Answer: Please see the answer to 3a and Minor Remark 5.

Remark: 3c. Were the input chemical shifts corrected for possible referencing errors? I wonder if using, e.g. a sequence-based prediction of the expected/neighbor-corrected random coil shift (e.g., POTENCI) might yield a more accurate "center" of the normal distribution for the expected shift given the solution pH and temperature.

Answer: We are thankful for the comment. Training and test data were simultaneously normalized in the original version of our work to correct referencing errors, but this fact was not mentioned in the manuscript. The following text has now been added at the end of the "Consistency of the training data" section:

"It is worth mentioning that chemical shift values of training and test proteins are normalized simultaneously using Pareto scaling to correct for possible referencing errors".

Remark: (4) On p. 8 the authors note that residues such as Glycine and Proline (and others with missing chemical shifts) were not used in the LDA classification procedure for alpha-synuclein. However, I worry that the authors might be discarding otherwise potentially useful data – for example, Gly residues are often used manually to make the first assignments given that XG/GX motifs in the sequence may be readily identified. And Pro residues have relatively unique chemical shifts that render them readily identified in the LDA classification shown in Figure 5B and 5C. In these plots, Gly is identified with 100% accuracy and Pro with 97% accuracy – thus, these two residues are highly useful and can be identified by LDA with high accuracy.

Answer: GLY and PRO data have been included in Figure 7.

Remark: 4a. Could the authors somehow include Gly and Pro in the calculation rather than excluding them?

Answer: See answer to remark 4.

Remark: 4b. Along the same lines as above, could the authors make use of non-Gly/Pro residues that have incomplete data? For example, in Figure 4 I note that the authors show

that CB is by far the most important for classification followed by HB and CA. The remaining chemical shifts are less important (< 0.1 relative importance). Thus, for residues that have at least CB HB and CA shifts, I would assume that these can still be used as input?

Answer: We are thankful for the comment. We do make use of residues with incomplete data, and this was stated in the following text in the Results sections:

“Some spin systems in the testing set may be incomplete, that is to say, lacking certain chemical shifts; this is usually the case for the residue preceding the formed chain of spin systems. To classify such spin systems we used a separate, restricted training set consisting of those chemical shifts that are present.”

Furthermore, Figure 7 has now been modified to include glycines and prolines, which have missing chemical shifts. In the same line, Figure 8 shows how the preceding residue and the last residue of the chain are accurately classified by LDA (and they are always missing one or more chemical shifts).

Remark: 5. Finally, given that the most important shifts for the LDA classification are CB and HB, and thus side-chain shifts, I wonder if the authors considered using C(CO)NH or H(CCO)NH spectra? Was there a particular reason to choose the 4D HabCab(CO)NH spectrum over those mentioned above? I ask because the C(CO)NH and H(CCO)NH spectra would not only provide the highly diagnostic CB and HB shifts, but also even more diagnostic shifts for residues that extend beyond CB/HB.

Answer: The proposed experiments, being 3D ones, provide worse resolution and thus are more difficult to interpret (i.e. to identify carbon and proton chemical shifts of the same residue). Information on further side-chain chemical shifts is of course valuable, but it also requires more difficult analysis in order to recognize which peak corresponds to which nucleus (alpha, beta, gamma etc.). The HabCab(CO)NH provides in our opinion the highest amount of information being also easy and fast to analyze.

Remark: For example, I note that Fig 5 shows that the LDA classification yields confidence values below 80% for Arg, Cys, Gln, Glu, Met, Phe, Trp, and Tyr residues. All of these except Cys would be very readily resolved with access to spins beyond HB/CB. I note that the authors have published an elegant 5D HC(CC-TOCSY)CONH that would provide access to some of these shifts.

Answer: Please see the answer to the remark above.

Remark: If the problem lies with poor access to chemical shift statistics of IDPs for spins beyond HB/CB, then that is indeed a fundamental problem. But given that the LDA classifer performed so well with only 18 proteins as training input, I would hope that there are at least 18 IDPs that have complete 1H and 13C side-chain assignments to enable LDA classification with this expanded benchmark for side-chain chemical shifts.

Answer: Please see the answer to the remark above.

Minor comments

Remark: 1. “alfa-synuclein” should be “alpha-synuclein” or better yet “α-synuclein”

Answer: Corrected

Remark: 2. p. 5 -- BMRB ID 6869 appears to be a folded protein? The web page with this ID is associated with the “solution structure of the C-Terminal 14 kDa Domain of the tau subunit from Escherichia coli DNA Polymerase III”

Answer: We are grateful for pointing this out (see also general remark #3 above). Indeed this protein got to the training data by accident. We removed it from the training set in the revised version.

Remark: 3. line 228, the “Section” number is missing

Answer: Corrected

Remark: 4. line 207, I would think that a citation should go here to e.g. some of the chemical-shift based work by the Bax group on TALOS or CS-Rosetta.

Answer: We are thankful for the suggestion. However, the mentioned programs aim at structure prediction based on chemical shifts, which can be done based on the deviation of particular chemical shifts of structured proteins from random coil values. On the other hand, the presented LDA method is based on the fact that for IDPs chemical shift values don’t deviate from the average in a systematic manner. To support our claim we chose to cite BMRB, since it is known from statistics that the mentioned chemical shifts are the most characteristic.

Remark: 5. why is the spread in Figure 4 between 98+% and 78+%? Is this due to an underlying AA compositional bias? Do all proteins all have the same AA distribution? It would be interesting to see a correlation between the accuracy and the AA diversity for each of the proteins in the training set. Having access to such information would enable the user to predict a priori how well the LDA approach will work on their system of interest based on the underlying AA composition. It may be important to re-train on a larger database in the future if certain AAs are poorly represented (e.g. aromatics and hydrophobics).

Answer: We appreciate the observation, and we understand the reviewer is talking about Figure 3 and not 4. The following Figure shows our attempts at trying to find a correlation between the accuracy of LDA and AA distribution:

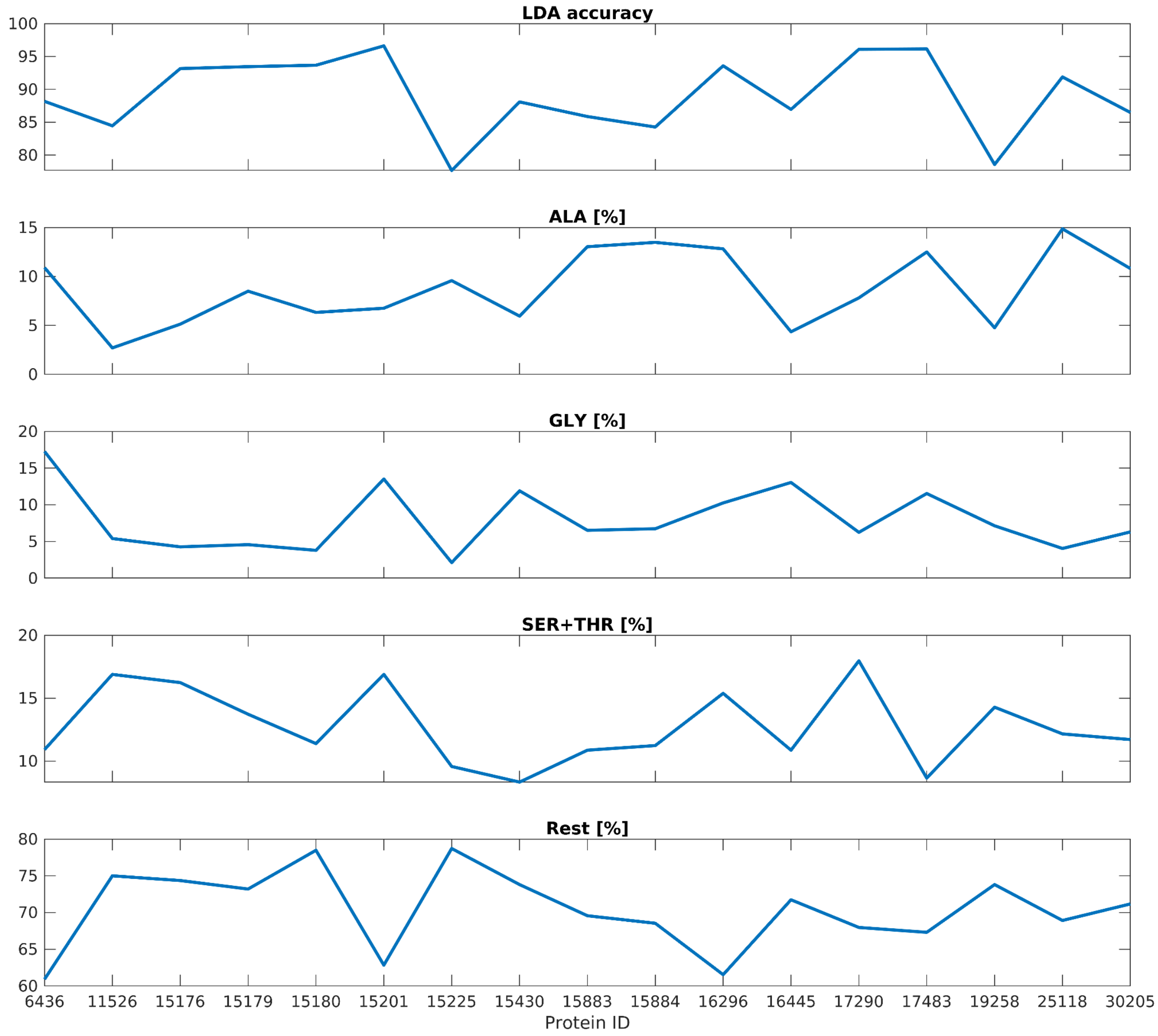

AA types were divided into 4 groups: alanine, glycine, serine + threonine and all others. The values are given in the percentage of each group present in each protein used for training the LDA algorithm. At first sight, the alanines group seems to hold some degree of correlation with LDA accuracy, but after doing the linear fitting, we found the R2 to be 0.0145. The coefficients of determination for linear fits of glycines, serine + threonine and all others are 0.0454, 0.097 and 0.1854, respectively. All these values are too low to be considered correlations. We also tried to find correlations between LDA accuracy and disorder level (as predicted by IUPred3), STD of amide proton's chemical shifts and an AA grouping consisting of arginine, asparagine, cysteine, glutamine, glutamic acid, histidine, lysine, methionine, proline, tryptophan, tyrosine and valine. The Figure below shows the results.

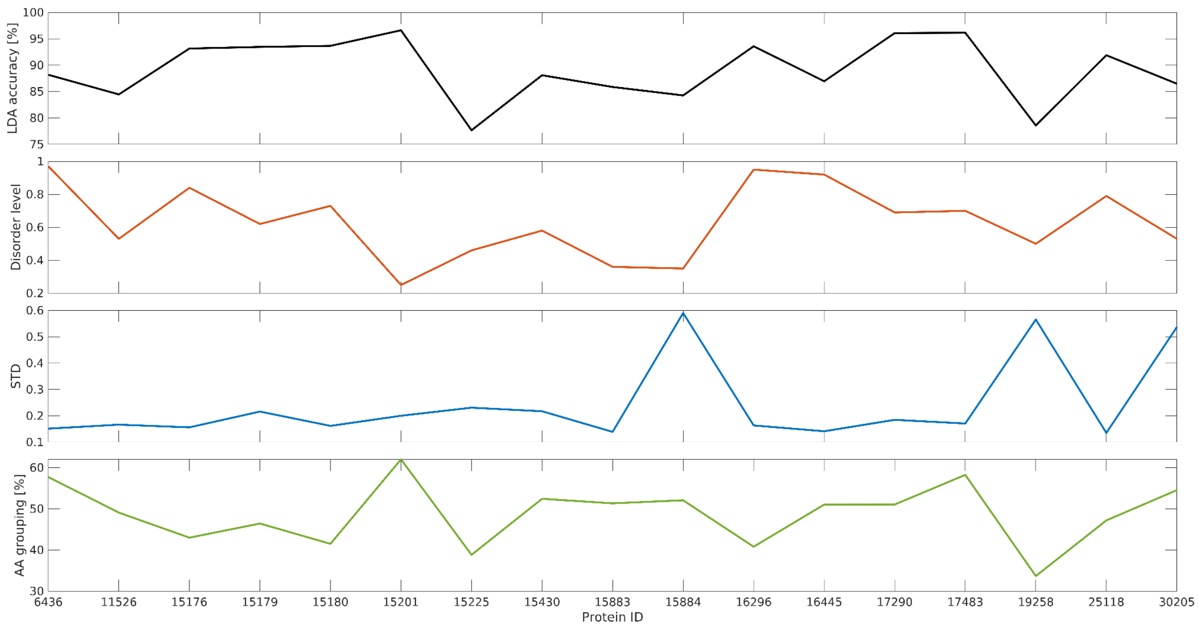

The R2 squares for the mentioned parameters and AA grouping are 0.1045, 0.2565 and 0.1696, respectively. All these low correlations point towards the "hidden patterns" mentioned in the title of our manuscript: it is not possible to single out one or even a handful of parameters that will accurately correlate with amino acid classification. This is why a sophisticated classification algorithm that will consider all chemical shifts and AA types at once is needed to solve the problem.

Still, Figure 3 has been modified to show the AA distribution of each protein in the test set. The readers should now be able to recognize that there is no correlation between LDA accuracy and AA distribution.

Remark: 6. For table 1, I guess the acq parameters for directly-detected 1H (t3 or t4) are the same and hence omitted? And I guess that ni = ni + ni2 (+ni3)?

Answer: Yes, all the direct-dimension parameters are identical and set to their default values. ni is the number of hypercomplex points of all indirect dimensions together (in the case of NUS experiments ni2, ni3 etc. are typically not defined).

Remark: 7. In a typical manual analysis of NMR data, it is usually trivial to identify and assign the XG/GX, XA/AX, XS/SX, and XT/TX spin systems, as the authors even write in their introduction. Thus, would it be possible to enable the user to manually "fix" specific assignments before running the calculation? You might envision that including e.g. the first 25% of assignments (G, A, S, T-containing spin systems) that are usually trivial to obtain might improve the LDA classification by having this starting point. I don't know if it's possible to do this though.

Answer: Please see our General Remark 2 (at the beginning of the Response to Reviewers).

Remark: 8. I commend the authors for making their data available on Zenodo. I downloaded the HNCO and HabCab(CO)NH spectra and looked at them in Sparky. I was surprised that

the HNCO contained so much noise; was it properly reconstructed? The HabCab(CO)NH spectrum on the other hand was very clean with minimal artifacts.

Answer: The NUS signal of HNCO was not reconstructed at all; we used direct FT of the NUS data to obtain the spectrum. Although such an approach is fully sufficient for 3D spectrum with such a low dynamic range of peak intensities (like HNCO), in response to the Reviewer’s comment, we also performed the artefact-cleaning procedure described in 10.1016/j.jmr.2007.08.005 and uploaded the spectrum to zenodo (new DOI: 10.5281/zenodo.7032142).
